# *Legionella*: A Promising Supplementary Indicator of Microbial Drinking Water Quality in Municipal Engineered Water Systems

**DOI:** 10.3389/fenvs.2021.684319

**Published:** 2021-11-10

**Authors:** Chiqian Zhang, Jingrang Lu

**Affiliations:** 1 Pegasus Technical Services, Inc., Cincinnati, OH, United States,; 2 Office of Research and Development, United States Environmental Protection Agency, Cincinnati, OH, United States

**Keywords:** *Legionella pneumophila*, *Mycobacterium*, *Pseudomonas*, drinking water distribution systems, premise plumbing, public health

## Abstract

Opportunistic pathogens (OPs) are natural inhabitants and the predominant disease causative biotic agents in municipal engineered water systems (EWSs). In EWSs, OPs occur at high frequencies and concentrations, cause drinking-water-related disease outbreaks, and are a major factor threatening public health. Therefore, the prevalence of OPs in EWSs represents microbial drinking water quality. Closely or routinely monitoring the dynamics of OPs in municipal EWSs is thus critical to ensuring drinking water quality and protecting public health. Monitoring the dynamics of conventional (fecal) indicators (e.g., total coliforms, fecal coliforms, and *Escherichia coli*) is the customary or even exclusive means of assessing microbial drinking water quality. However, those indicators infer only fecal contamination due to treatment (e.g., disinfection within water utilities) failure and EWS infrastructure issues (e.g., water main breaks and infiltration), whereas OPs are not contaminants in drinking water. In addition, those indicators appear in EWSs at low concentrations (often absent in well-maintained EWSs) and are uncorrelated with OPs. For instance, conventional indicators decay, while OPs regrow with increasing hydraulic residence time. As a result, conventional indicators are poor indicators of OPs (the major aspect of microbial drinking water quality) in EWSs. An additional or supplementary indicator that can well infer the prevalence of OPs in EWSs is highly needed. This systematic review argues that *Legionella* as a dominant OP-containing genus and natural inhabitant in EWSs is a promising candidate for such a supplementary indicator. Through comprehensively comparing the behavior (i.e., occurrence, growth and regrowth, spatiotemporal variations in concentrations, resistance to disinfectant residuals, and responses to physicochemical water quality parameters) of major OPs (e.g., *Legionella* especially *L*. *pneumophila*, *Mycobacterium*, and *Pseudomonas* especially *P*. *aeruginosa*), this review proves that *Legionella* is a promising supplementary indicator for the prevalence of OPs in EWSs while other OPs lack this indication feature. *Legionella* as a dominant natural inhabitant in EWSs occurs frequently, has a high concentration, and correlates with more microbial and physicochemical water quality parameters than other common OPs. *Legionella* and OPs in EWSs share multiple key features such as high disinfectant resistance, biofilm formation, proliferation within amoebae, and significant spatiotemporal variations in concentrations. Therefore, the presence and concentration of *Legionella* well indicate the presence and concentrations of OPs (especially *L*. *pneumophila*) and microbial drinking water quality in EWSs. In addition, *Legionella* concentration indicates the efficacies of disinfectant residuals in EWSs. Furthermore, with the development of modern *Legionella* quantification methods (especially quantitative polymerase chain reactions), monitoring *Legionella* in ESWs is becoming easier, more affordable, and less labor-intensive. Those features make *Legionella* a proper supplementary indicator for microbial drinking water quality (especially the prevalence of OPs) in EWSs. Water authorities may use *Legionella* and conventional indicators in combination to more comprehensively assess microbial drinking water quality in municipal EWSs. Future work should further explore the indication role of *Legionella* in EWSs and propose drinking water *Legionella* concentration limits that indicate serious public health effects and require enhanced treatment (e.g., booster disinfection).

## INTRODUCTION

Microbial drinking water quality (i.e., the presence and concentrations of microbes) in municipal engineered water systems (EWSs, including drinking water distribution systems or DWDSs and building premise plumbing systems or PSSs) strongly affects public health (Ashbolt, 2015a; Zhang and Lu, 2021). Pathogenetic microbes in EWSs may infect end consumers and cause life-threatening diseases or disease outbreaks. Microbes enter municipal EWSs with finished water from treatment plants. Drinking water treatment facilities effectively remove and inactivate microbes, especially pathogens, with disinfection (e.g., chlorination, chloramination, and ozonation) and other treatment barriers (e.g., filtration) before discharging finished water to DWDSs (McGuire, 2006; Okpara et al., 2011; Zhang and Liu, 2019). However, residual microbes in finished water could be still active or viable [or viable-but-non-culturable (VBNC)] and enter DWDSs (Haas, 1999; Schoenen, 2002; Liu et al., 2009; Marciano-Cabral et al., 2010; Falkinham et al., 2015a; Lin et al., 2017; Chen et al., 2018; Guo et al., 2021). Microbes also invade EWSs because of deficiencies in design and operations of the systems, installation and maintenance of water mains and distal pipes, and intrusion during cross-connections (actual or potential connections between potable water supplies and non-potable sources), back-pressure (occurs when the supply pressure is lower than the local pipe pressure; a type of backflow), and back-siphonage (due to local negative pressure transients or a vacuum effect; another type of backflow) (Herrick, 1997; Payment, 1999; LeChevallier et al., 2003; Lee and Schwab, 2005; Makris et al., 2014; Beer et al., 2015; Gerba and Pepper, 2015; Lu et al., 2016; Leslie et al., 2021).

Certain microbes after entering or invading municipal EWSs survive, (re)grow, and become natural inhabitants of drinking water. Most natural inhabitants in EWSs are harmless to humans. However, an important group of natural inhabitants in EWSs, water-based opportunistic pathogens (OPs), is the leading cause of drinking-water-based disease outbreaks, significantly threatens public health, and causes serious social-economic losses (Wang et al., 2013; Whiley et al., 2014b; Ashbolt, 2015b; Beer et al., 2015; Falkinham et al., 2015b; Benedict et al., 2017; Liu et al., 2017; El-Chakhtoura et al., 2018; Cullom et al., 2020). For instance, water-based nontuberculous mycobacteria (NTM) infections annually cause over 16,000 hospitalizations and a total hospitalization cost of over 426 million United States (US) dollars in the US (Collier et al., 2012). In addition, OPs survive and (re)grow in EWSs (Hwang et al., 2012; El-Chakhtoura et al., 2018; Douterelo et al., 2019; Zhang and Liu, 2019) even in the presence of disinfectant residuals (Haas, 1999; LeChevallier, 1999; van der Kooij, 2003; Zhang et al., 2019; Zhang et al., 2021a) and under high-shear turbulent flow conditions (Lehtola et al., 2007). Furthermore, conventional water quality indicators (e.g., total and fecal coliforms, thermotolerant coliforms, *Escherichia coli*, enterococci, and fecal streptococci) and fecal contaminants (i.e., enteric and primary pathogens) that commonly exist in (contaminated) natural waterbodies occur in EWSs at low frequencies and concentrations and are often absent from well-maintained systems (Falkinham, 2020; Zhang and Lu, 2021). Therefore, the presence and concentrations of OPs are the major aspect of microbial water quality in municipal EWSs. Because OPs in drinking water frequently cause disease outbreaks, monitoring the dynamics of OPs in municipal EWSs is an important and urgent task for the water industry and governmental agencies to deeply understand microbial drinking water quality and to better protect public health.

Examining all or dominant (pathogenic) microbes to fully understand microbial drinking water quality in EWSs is technically intense, expensive, time consuming, and often impractical and unnecessary (Gerba, 2015). Therefore, water utilities and public health agencies in the US and many other countries assess microbial drinking water quality mainly or even exclusively through quantifying conventional indicator microbes (i.e., fecal indicators). Fecal indicators infer fecal contamination from warm-blooded animals and the presence of enteric/primary pathogens due to treatment system failure, water pipe breaks, cross-connections, and backflow (Cohen and Shuval, 1973; Edberg et al., 2000; Ashbolt et al., 2001; Lee and Schwab, 2005; Tallon et al., 2005; Gerba, 2015; Lu et al., 2016). Examining fecal indicators using traditional culture-based methods (e.g., the Colilert^™^ Test for both total coliforms and *E*. *coli*) is straightforward and cost effective. However, as discussed above, conventional fecal indicators and fecal contaminants (i.e., enteric pathogens) have low concentrations and are often absent in EWSs. Fecal contaminants and indicators are thus not the major aspect of microbial drinking water quality, especially in (developed) countries where secondary disinfection is mandatory (e.g., the US) and where municipal EWSs are well-maintained and clean (e.g., the Netherlands) (Smeets et al., 2009; Zhang and Liu, 2019; Zhang and Lu, 2021). In addition, fecal indicators behave distinctively from OPs in EWSs (Falkinham et al., 2015b; Falkinham, 2015; Lu et al., 2016; Wang et al., 2017; Perrin et al., 2019a; Zhang and Lu, 2021). For instance, the concentrations of OPs generally increase, while fecal indicators decay with increasing hydraulic residence time (HRT) of distributed water (Falkinham, 2020; Zhang and Lu, 2021). OPs have high resistance to disinfectant residuals (and thus have high concentrations), while fecal indicators are susceptible to disinfectant residuals (and thus have low concentrations or are even absent) in EWSs (Falkinham, 2015). Therefore, conventional fecal indicators do not indicate OPs (the major aspect of microbial drinking water quality) in EWSs (Lehtola et al., 2007; LeChevallier, 2019), and a supplementary or additional indicator that specifically infers the prevalence of OPs is highly needed. To date, a microorganism indicating the presence and concentrations of OPs in municipal EWSs has not been fully developed, evaluated, and validated. To the best of our knowledge, most countries do not require a routine minoring of OPs in municipal EWSs using an indicator microorganism.

This comprehensive literature review aims to purpose an appropriate indicator microorganism that can infer the presence and concentrations of water-based OPs (the major aspect of microbial drinking water quality) in municipal EWSs. We first explain why conventional fecal indicators fail to indicate OPs or microbial drinking water quality in EWSs and why an indicator of OPs is highly needed. We then propose novel critical criteria for selecting an appropriate indicator of the presence and concentrations of OPs in drinking water. We argue that selecting such a supplementary indicator from common OPs in EWSs is appropriate. We finally comprehensively compare the behavior (i.e., occurrence, growth and regrowth, spatiotemporal variations in concentrations, resistance to disinfectant residuals, and responses to physicochemical water quality parameters) of major OPs (i.e., *Legionella* especially *L*. *pneumophila*, *Mycobacterium*, and *Pseudomonas* especially *P*. *aeruginosa*). The comparison proves that *Legionella* is a promising supplementary indicator for the presence and concentrations of OPs in EWSs while other OPs do not have this indication feature. This comprehensive literature review for the first time proposes and confirms an appropriate indicator microorganism (*Legionella*) that well infers water-based OPs and reflects microbial drinking water quality in municipal EWSs, adding new insights into the current literature. This review also systematically summarizes the behavior of *Legionella* and other common OPs in municipal drinking water, whereas such information is meaningful to developing more effective OP control strategies and promoting public health.

## FECAL INDICATORS FAIL TO INDICATE OPPORTUNISTIC PATHOGENS IN MUNICIPAL ENGINEERED WATER SYSTEMS

Examining conventional fecal indicators is the most common practice worldwide evaluating drinking water quality in municipal EWSs. However, fecal indicators indicate only the presence of enteric pathogens and/or fecal contaminants (mainly due to treatment failure in water utilities) and cannot infer the presence and concentrations of water-based OPs (the major aspect of microbial drinking water quality). Fecal indicators fail to indicate OPs because of three reasons. First, fecal indicators and OPs in municipal EWSs have distinct microbial community compositions. Fecal indicators mainly include *E*. *coli*, *Bacteroides* (more common than *E*. *coli* in the gastrointestinal tract of humans; a good indicator for recent fecal contamination), fecal coliforms, and fecal streptococci (enterococci) (Gerba, 2015). Common water-based OPs in EWSs are *Legionella* (especially *L*. *pneumophila*), *Pseudomonas* (especially *P*. *aeruginosa*), *Mycobacterium* or NTM (especially *M*. *avium* and *Mycobacterium avium* complex or MAC; MAC includes mainly two closely related species, *M*. *avium* and *M*. *intracellulare*), *Aeromonas*, *Acinetobacter baumannii*, *Flavobacterium*, *Acanthamoeba*, *Vermamoeba vermiformis* (formerly *Hartmannella vermiformis*), and *Naegleria fowleri* (Rusin et al., 1997; Ford, 1999; Falkinham et al., 2001; van der Kooij, 2003; Pryor et al., 2004; September et al., 2004; Lau and Ashbolt, 2009; ldi et al., 2010; Wingender and Flemming, 2011; Wang et al., 2012a; van der Wielen and van der Kooij, 2013; Delafont et al., 2014; Wang et al., 2014; Ashbolt, 2015b; Falkinham et al., 2015b; Boyle et al., 2015; Lu et al., 2016; Kim et al., 2017; Delafont et al., 2018; Perrin et al., 2019b; Dai et al., 2019; Isaac and Sherchan, 2019; Zhang et al., 2021b; Zhang and Lu, 2021). The general term “water-based” or “water-related” is more appropriate than “water-borne” to describe OPs in EWSs (Ashbolt, 2015a).

**FIGURE 1 |:**
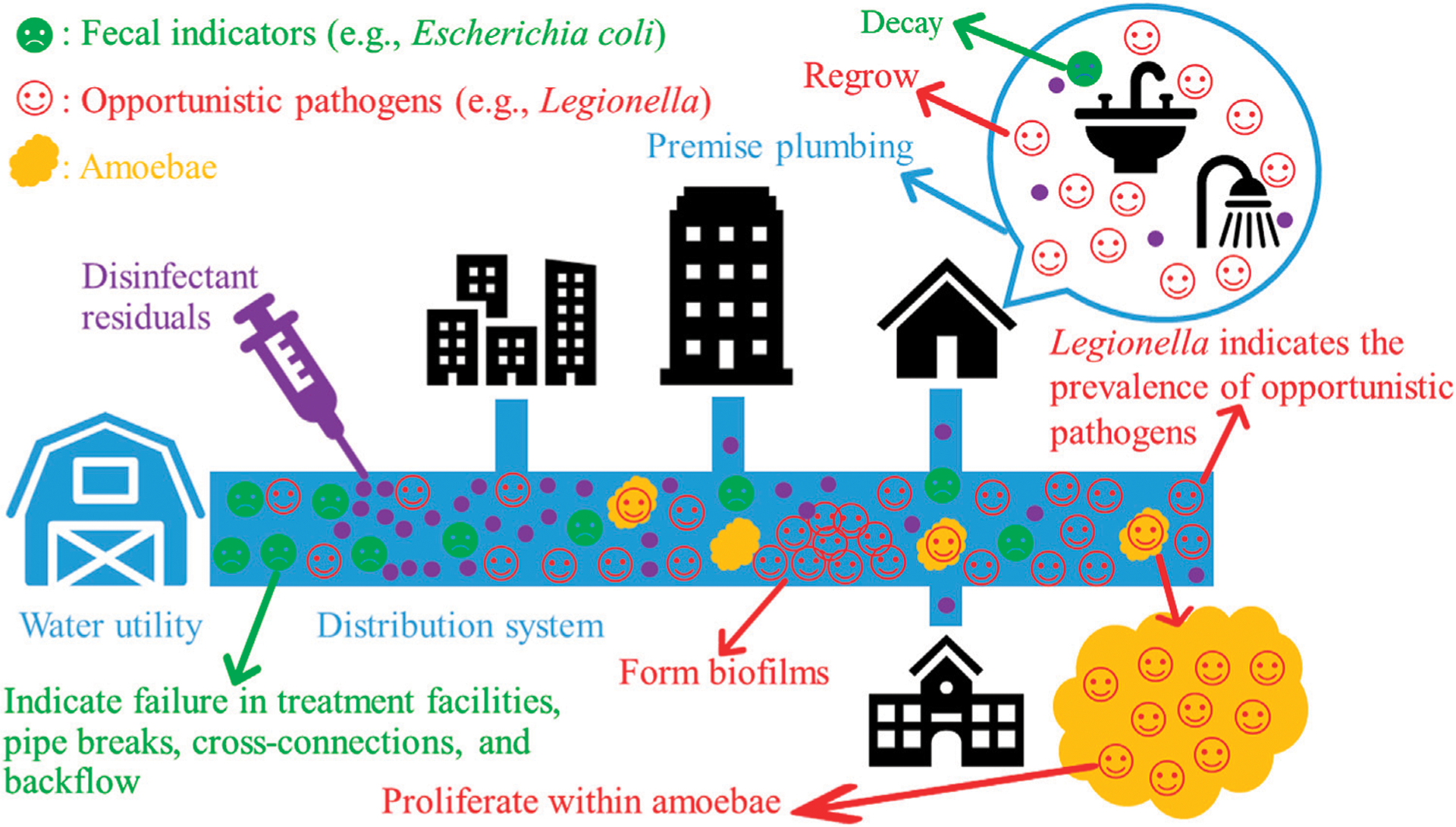
The decay of conventional fecal indicators and the (re)growth of opportunistic pathogens (OPs) along municipal engineered water systems (EWSs). Bacterial OPs often proliferate within their amoeba hosts and form biofilms in EWSs. Drawn using PowerPoint (version 2102, Microsoft Office Professional Plus 2019) (Microsoft Corporation, Redmond, Washington, USA).

Second, the unique environment (e.g., high surface to volume ratio and low organic matter concentration) of EWSs favors the persistence and (re)growth of water-based OPs over fecal indicators (Falkinham et al., 2015b). Common OPs, such as *Legionella*, *Mycobacterium*, and *Pseudomonas* (re)grow well in such an environment (Ribas et al., 2000; Declerck et al., 2009; van der Wielen and van der Kooij, 2013; Lu et al., 2016; Bédard et al., 2019) (Figure 1). Because of their significant (re)growth, the concentrations of OPs increase with the HRT of distributed water along EWSs (Falkinham, 2015). For instance, the concentrations of *Legionella*, *Mycobacterium*, *P*. *aeruginosa*, *Acanthamoeba*, and *V*. *vermiformis* downstream in a DWDS in the US were generally greater than those at the entry point of the system (Lu et al., 2016). A recent study similarly found that the concentrations of four common OPs (*Legionella*, *Mycobacterium*, *Pseudomonas*, and *V*. *vermiformis*) increased with the HRT of distributed water in four full-scale chloraminated DWDSs in the US (Zhang et al., 2021b). OPs are thus native inhabitants of EWSs and occur more frequently and at greater concentrations downstream in the systems (Falkinham et al., 2015b; Wang et al., 2017). By contrast, the most common fecal indicator *E*. *coli* persists but does not substantially regrow in EWSs (Edberg et al., 2000; Abberton et al., 2016; Lu et al., 2016). In general, the concentrations of *E*. *coli* and other fecal indicators fall when drinking water moves along EWSs because of dilution and non-growth (Falkinham, 2015) (Figure 1).

Third, water-based OPs are generally (much) more resistant to disinfectant residuals (which are commonly dosed to distribution systems) than fecal indicators in EWSs (Sobsey, 1989; Payment, 1999; Falkinham, 2015; Falkinham, 2020; Zhang and Lu, 2021). For instance, the *C*·*t*_99.9%_ [the product of disinfectant concentration *C* (mg·L^−1^) and contact time *t* (min) to kill 99.9% of cells] value of free chlorine for medium-grown *E*. *coli* was 0.09 mg·min·L^−1^ at 23 °C (Taylor et al., 2000). The *C*·*t*_99.9%_ values of free chlorine for medium-grown (51 to 204 mg·min·L^−1^ at 23 °C) and drinking-water-grown (551 to 1,552 mg·min·L^−1^ at 23 °C) *M*. *avium* were significantly greater. Similarly, planktonic *P*. *aeruginosa* is more resistant than planktonic *E*. *coli* to free chlorine, chloramines, and ozone (Ross et al., 1976). *Legionella* is also more resistant to disinfectants than *E*. *coli* and other coliform bacteria (Kim et al., 2002). For example, *L*. *pneumophila* was more resistant to ozone than *E*. *coli* in a continuous flow reactor (Botzenhart et al., 1993). Residuals of free oxidants (an electrochemical disinfectant) in the range from 4.05 to 19.35 mg·min·L^−1^ at 21 °C killed 95.9% to 98.6% of *L*. *pneumophila* in tap water but eradicated *E*. *coli* (Delaedt et al., 2008). In addition, exposure of clinical isolates of *A*. *baumannii* to free chlorine (4 mg·L^−1^) for 2 min in deionized water did not significantly reduce their culturable cell counts (*p* > 0.05) (Karumathil et al., 2014). On the other hand, certain studies showed that *L*. *pneumophila* is more sensitive to disinfectants than *E*. *coli*. For instance, a study found that planktonic *L*. *pneumophila* serogroup 1 (*C*·*t*_99%_ value 15 mg·min·L^−1^) was more susceptible to monochloramine than planktonic *E*. *coli* (*C*·*t*_99%_ value 37 mg·min·L^−1^) in the bulk water of a laboratory-scale plumbing system (Cunliffe, 1990). Another study found that *L*. *pneumophila* was more susceptible to chlorine dioxide than *E*. *coli* (Botzenhart et al., 1993). Future studies should more comprehensively compare the susceptibilities between OPs and fecal indicators to disinfectants or disinfectant residuals.

Because the environment of EWSs favors the (re)growth of OPs and OPs are resistant to disinfectant residuals, OPs (re)grow better and occur more frequently and at much greater concentrations than conventional fecal indicators in EWSs (Figure 1). For instance, culturable *E*. *coli* survived only 5 d in the biofilms and 9 d in the bulk water of a drinking water Propella biofilm reactor, but *M*. *avium* (more than 4 weeks) and *L*. *pneumophila* (more than 2 weeks) survived much longer in the biofilms and water (Lehtola et al., 2007). Similarly, *L*. *pneumophila* and *P*. *aeruginosa* spiked to flow-through reactors (continuously perfused with tap water) entered and persisted for weeks in biofilms on rubber/plastic plumbing materials, but spiked *Enterobacter nimipressuralis* [a coliform species (Kämpfer et al., 2008)] was absent from the biofilms (Moritz et al., 2010). In two full-scale DWDSs in South Australia, *E*. *coli* and total coliforms were absent from the bulk water, but *Legionella*, *L*. *pneumophila*, and MAC were present in the water throughout the year (Whiley et al., 2014a). In 10 full-scale DWDSs in the US, *L*. *pneumophila* occurred in 14 out of 573 bulk water samples, while total coliforms and *E*. *coli* were absent in those 573 samples (LeChevallier, 2019). In hot tap water from six buildings on a university campus in Poland, *E*. *coli* had low concentrations [approximately 0 genome copy number per liter (GCN·L^−1^; GCN also stands for “gene copy number” in this review) or below the limit of detection], whereas *Legionella* (4.52 × 10^3^ to 1.56 × 10^4^ GCN·L^−1^ for positive sampling sites) and *L*. *pneumophila* (0.98 × 10^3^ to 1.20 × 10^4^ GCN·L^−1^ for positive sampling sites) frequently occurred at much greater concentrations (Wolf-Baca and Siedlecka, 2019). In four full-scale reclaimed water distribution systems in the US, *Legionella*, *Mycobacterium*, *Pseudomonas*, and *Aeromonas* occurred more frequently and generally had much greater concentrations than total coliforms, fecal coliforms, *E*. *coli*, and enterococci (Jjemba et al., 2010).

The three reasons discussed above confirm that water-based OPs and conventional fecal indicators behave differently in drinking water with distinct community compositions, (re) growth patterns, and susceptibilities to disinfectant residuals. Therefore, fecal indicators are uncorrelated with and fail to indicate OPs in EWSs (ldi et al., 2010; Falkinham, 2015; Wang et al., 2017; Perrin et al., 2019a; Isaac and Sherchan, 2019). Indeed, a human fecal indicator *Bacteroides* and an enteric pathogen *E*. *coli* O157:H7 were absent from the bulk water of a DWDS in a metropolitan area in the US (Lu et al., 2016), the sediments of 18 drinking water storage tanks (DWSTs) across 10 states in the US (Lu et al., 2015), and the tap and shower water from a simulated house premise plumbing in the US (Lu et al., 2017). However, representative OPs including *Mycobacterium*, *Legionella*, *P*. *aeruginosa*, *Acanthamoeba*, and *V*. *vermiformis* frequently occurred at those sites at relatively high concentrations. In addition, in the hot water from hot water distal outlets in 28 commercial buildings in the US, the concentration of HPC bacteria was uncorrelated with *Legionella* positivity (*p* 0.788) and explained only 0.68% of the variation in *Legionella* positivity (Pierre et al., 2019). In the shower water from a US hospital and cooling water from pilot-scale cooling towers, the concentrations of both HPC bacteria and adenosine triphosphate failed to accurately predict *Legionella* positivity or colonization (Duda et al., 2015).

The current routine examination of microbial drinking water quality by detecting conventional fecal indicators fails to assess the negative health effects of water-based OPs in EWSs and is thus biased. First, quantifying fecal indicators is the main or even exclusive means of examining microbial drinking water quality in municipal EWSs (Edberg et al., 2000; Gerba, 2015; Wen et al., 2020). To the best of our knowledge, the revised Drinking Water Directive of the European Union (EU) is the only regulation that requires monitoring *Legionella* in DWDSs for risk assessment [parametric value: < 1,000 colony forming units (CFU) per liter] (The Council of the EU, 2020). Most current drinking water standards and regulations enforce only the quantification of fecal indicators. For instance, through the Safe Drinking Water Act, the US Environmental Protection Agency (US EPA) has established the National Primary Drinking Water Regulations, which require using total coliforms (including fecal coliforms and *E*. *coli*) as an indicator of potentially harmful bacteria and set an enforceable Maximum Contaminant Level (MLC) for total coliforms in drinking water to be 5.0% (i.e., total-coliform-positive samples in a month being less than 5.0%) (US EPA, 1989). The same Regulations set a non-enforceable Maximum Contaminant Level Goal (MCLG) for both total coliforms and *Legionella* to be 0 mg·L^−1^ [*sic*] in drinking water. Similarly, the EU sets the maximum number of *E*. *coli*, coliform bacteria, and intestinal enterococci to be 0 per 100 mL of tap water (The Council of the EU, 2020). As discussed above, fecal indicators are uncorrelated with OPs in EWSs, and the presence, absence, and/or concentrations of fecal indicators in drinking water cannot reflect the negative public health effects of water-based OPs. Second, despite the critical role of EWSs in maintaining drinking water quality, most standards regulate only the levels of fecal indicators in the water entering DWDSs (i.e., finished water or plant effluent) and leaving PPSs (e.g., tap water). In the US, the Revised Total Coliform Rule is the only regulation that requires quantifying fecal indicators (i.e., total coliforms and *E*. *coli*) throughout DWDSs (US EPA, 2013). Third, even though *Legionella* frequently occurs in municipal EWSs and causes drinking-water-based disease (i.e., Legionnaires’ disease) outbreaks, routine monitoring of *Legionella* in such systems is rare (Hamilton et al., 2019). Some researchers even inadequately argued for the removal of the requirement for routine monitoring of *Legionella* in EWSs from all drinking water guidelines (Whiley, 2017). At least, at distal sites of PPSs in hospitals, water authorities or medical personnel need to routinely monitor *Legionella* to protect the health of patients (Baron et al., 2019). In summary, the routine examination of microbial drinking water quality by detecting fecal indicators unreasonably neglects OPs, which are the major disease causative biotic agent in municipal drinking water. The primary reason for the negligence is that an indicator inferring the presence and concentrations of water-based OPs (the major aspect of microbial drinking water quality) in EWSs is not fully developed, evaluated, and validated. Therefore, we dedicate this literature review to proposing an appropriate indicator (i.e., *Legionella*) of OPs that can better reflect microbial drinking water quality in municipal EWSs. Water utilities and authorities may use *Legionella* as a supplementary indicator in conjunction with conventional fecal indicators to more comprehensively evaluate microbial drinking water quality and to better protect public health (Zhang et al., 2021b).

## NOVEL CRITERIA FOR SELECTING AN APPROPRIATE INDICATOR OF OPPORTUNISTIC PATHOGENS IN MUNICIPAL ENGINEERED WATER SYSTEMS

Scientists have developed multiple criteria for selecting indicator microorganisms to reflect microbial water quality (Gerba, 2015; Wen et al., 2020). Existing criteria are meaningful for selecting indicators of fecal contamination and/or enteric pathogens in drinking water, surface water, recreational water, and wastewater (Gerba, 2015). However, since fecal indicators and OPs in drinking water behave distinctly (Falkinham et al., 2015b; Wang et al., 2017), not all those existing criteria are suitable for selecting an indicator of OPs in municipal EWSs. We need to revise those criteria to select an appropriate indicator microorganism for the presence and concentrations of OPs (the major aspect of microbial drinking water quality) in municipal EWSs. Such revised criteria are missing in the literature. We propose the following novel critical criteria to select an appropriate indicator microorganism of water-based OPs in EWSs:

- The indicator is one important or dominant member of the natural inhabitants (e.g., OPs) of drinking water. This property of the indicator ensures that the presence and concentration of the indicator suggest the presence and concentration of OPs as a whole group.
- The indicator occurs in municipal EWSs at a high frequency and concentration and can be feasibly examined through multiple approaches. This property of the indicator makes its detection and quantification easy, flexible (i.e., can be assayed by different methods), and affordable.
- The presence and concentration of the indicator in EWSs closely correlate with the presence and concentrations of water-based OPs and other critical physicochemical and microbial drinking water quality parameters. This property of the indicator ensures that when the indicator occurs at a high (or low) concentration, the overall concentration of OPs and the public health risks of the drinking water are likely high (or low).
- The occurrence and concentration of the indicator indicate the efficacies of disinfectant residuals. This property links the presence and concentration of the indicator to the efficacy of secondary disinfection (a critical treatment barrier). Therefore, a high frequency of occurrence and concentration of the indicator could suggest drinking water quality deterioration (due to disinfectant residual decay and/or consumption) and the need for enhanced or additional treatment (such as booster disinfection and distribution system cleaning/upgrade).

## THE POTENTIALS OF NATURAL INHABITANTS TO INDICATE OPPORTUNISTIC PATHOGENS IN MUNICIPAL ENGINEERED WATER SYSTEMS

Conventional fecal indicators behave distinctly from and are uncorrelated with water-based OPs in municipal EWSs (Falkinham, 2020). Therefore, we need to select a novel indicator that can indicate the presence and concentrations of OPs in drinking water for more effective OP control and enhanced public health protection. This novel indicator should be selected from common OPs rather than from non-pathogenic natural inhabitants in municipal EWSs (Table 1). The occurrence and concentrations of non-pathogenic species (which are harmless to humans) do not correlate with the public health risks posed by OPs in drinking water. By contrast, a dominant OP could well indicate the health risks posed by OPs. That dominant OP is itself opportunistically pathogenic and poses public health risks. In addition, the presence and concentration of that dominant OP could well correlate with the presence and concentrations of other OPs because OPs in EWSs share many characteristics such as high resistance to disinfectant residuals, biofilm formation, and proliferation within amebae (Falkinham, 2015; Zhang and Lu, 2021). Therefore, this review selects a novel indicator of OPs from common OPs in municipal EWSs. On the basis of a comprehensive comparison among the behavior of dominant OPs (*Legionella*, *Mycobacterium*, and *Pseudomonas*) in drinking water, we conclude that *Legionella* is a promising indicator microorganism inferring the public health risks posed by water-based OPs in municipal EWSs while other OPs lack this indication feature (Zhang et al., 2021b).

Water-based OPs in EWSs have a diverse community with multiple bacterial and amoebal species, while the most common and dominant OPs are *Legionella* (especially *L*. *pneumophila*), *Mycobacterium* (especially NTM, *M*. *avium*, and MAC), and *Pseudomonas* (especially *P*. *aeruginosa*) (Falkinham, 2015; Falkinham, 2020; Lu et al., 2017; Lu et al., 2016; Lu et al., 2015; Zhang and Lu, 2021; Zhang et al., 2021b). Other OPs (such as certain amoebae) occur in drinking water at lower frequencies and/or lower concentrations and thus do not accurately suggest the overall health risks of OPs in municipal EWSs (Table 1). For instance, low concentrations of other OPs in EWSs do not imply that the public health risks of OPs in the drinking water are low, because the three dominant OPs could occur at high concentrations and have a high potential for infection of immunocompromised and immunocompetent populations. Amoebae in drinking water could have strong correlations with bacterial OPs such as *Legionella* and *Mycobacterium* (but not always) (Wang et al., 2012a; Wang et al., 2012b; Lu et al., 2016; Valciņa et al., 2019; Zhang et al., 2021b). The association between *Legionella* and its amoeba hosts mainly exists in pipe biofilms in municipal EWSs (Storey et al., 2004a; Declerck et al., 2007; Lau and Ashbolt, 2009; Thomas and Ashbolt, 2011; Wang et al., 2012a), whereas the detection and monitoring of *Legionella* are mainly for planktonic *Legionella* in bulk water. Therefore, the amoeba-*Legionella* relationships do not warrantee that amoebae are a good indicator of OPs in municipal EWSs. *Pseudomonas* (especially *P*. *aeruginosa*) as a common OP in EWSs have (much) lower concentrations and frequencies of occurrence than *Legionella* and *Mycobacterium* (Wang et al., 2012a; Lu et al., 2016; Lu et al., 2017; Zhang et al., 2021b). Therefore, the occurrence and concentration of *Pseudomonas* (especially *P*. *aeruginosa*) also do not fully reflect the health risks of OPs, and *Pseudomonas* (especially *P*. *aeruginosa*) is not a good indicator of OPs in municipal EWSs (Table 1).

By contrast, *Legionella* (especially *L*. *pneumophila*) and *Mycobacterium* (especially NTM, *M*. *avium*, and MAC) are the two most dominant OPs in municipal EWSs with comparably high frequencies of occurrence and concentrations (Wang et al., 2012a; Lu et al., 2016; Lu et al., 2017; Zhang et al., 2021b). *Legionella* (*L*. *pneumophila*) and *Mycobacterium* (*M*. *avium*) both have a sporadic occurrence nature in EWSs (Donohue et al., 2019). When either *Legionella* or *Mycobacterium* occurs at a high concentration, the overall concentration of OPs is high, and the corresponding drinking water poses significant public health risks, requiring enhanced treatment (e.g., booster disinfection) and distribution system cleaning or upgrade. Oppositely, when the concentration of either *Legionella* or *Mycobacterium* is low, the overall concentration of OPs would be low, and the corresponding drinking water would have a low risk of causing disease outbreaks. Therefore, either *Legionella* (especially *L*. *pneumophila*) or *Mycobacterium* (especially NTM, *M*. *avium*, and MAC) could be a good indicator of water-based OPs and their public health risks in municipal EWSs (Table 1).

Compared with *Mycobacterium*, *Legionella* could be a better indicator of water-based OPs in municipal EWSs. First, *Legionella* (especially *L*. *pneumophila*) is the first leading cause of drinking-water-based disease outbreaks (Beer et al., 2015; Benedict et al., 2017) and correlates with more OPs than *Mycobacterium*. For instance, in four full-scale chloraminated DWDSs in the US, the concentration of *Legionella* significantly and positively correlated with those of *Mycobacterium* (*p* 0.042), *Pseudomonas* (*p* < 0.001), and total OPs (i.e., the sum concentration of all four examined OPs; *p* < 0.001) (Zhang et al., 2021b). *Legionella* also had a nearly significant positive linear correlation with *V*. *vermiformis* (*p* 0.054). Therefore, the presence of *Legionella* at a relatively high concentration indicates a well-built growth condition for other bacterial OPs (e.g., *Mycobacterium* and *Pseudomonas* especially *P*. *aeruginosa*) and their amoeba hosts (e.g., *Acanthamoeba*, *N*. *fowleri*, and *V*. *vermiformis*) in municipal EWSs. By contrast, *Mycobacterium* had a significant positive linear correlation with only *Legionella* (*p* 0.042). Similarly, a multiple linear regression reveals that *Legionella* closely correlated with more physicochemical water quality parameters than *Mycobacterium* in the four chloraminated DWDSs (Zhang et al., 2021b). *Legionella* had significant linear correlations with water temperature (*p* 0.002), total chlorine residual concentration (*p* 0.012), free ammonia concentration (*p* 0.037), and total trihalomethane (THM) concentration (*p* 0.019) (negative correlations except for with free ammonia concentration). *Mycobacterium* had significant linear correlations with only total chlorine residual concentration (*p* < 0.001) and total haloacetic acid concentration (*p* 0.018). Since *Legionella* correlates with more OPs and more critical physicochemical water quality parameters than *Mycobacterium*, the presence and concentration of *Legionella* reflect better the public health risks posed by OPs and even general drinking water quality in municipal EWSs. Second, *Mycobacterium* (*M*. *avium*) has a much higher resistance to disinfectant residuals than *Legionella* (*L*. *pneumophila*) and is thus insensitive to changes in disinfectant residual levels and efficacies (Falkinham, 2015). Therefore, when disinfectant residuals are at levels effective enough in suppressing the (re) growth of OPs (as well as enteric pathogens and/or fecal contaminants) and ensuring a good drinking water quality, the concentration of *Mycobacterium* (*M*. *avium*) could be relatively high, and using *Mycobacterium* (*M*. *avium*) as an indicator of water-based OPs might overestimate the public health risks of OPs in drinking water. Third, among the many OPs in EWSs, *Legionella* (*L*. *pneumophila*) is the most widely known and studied (Falkinham, 2015). Numerous culture-dependent and culture-independent assays are available for *Legionella* (*L*. *pneumophila*) detection and quantification (Wang et al., 2017). With the fast development of modern molecular techniques (especially quantitative polymerase chain reactions or qPCRs), assessing the concentration and diversity of *Legionella* (*L*. *pneumophila*) in drinking water is becoming more straightforward, easier to handle, more affordable, and faster (Girones et al., 2010; Lu et al., 2015; Lu et al., 2016; Wolf-Baca and Siedlecka, 2019; Zhang et al., 2021b). In conclusion, we argue that among the many OPs in municipal EWSs, *Legionella* is the most appropriate indicator of the presence and concentrations OPs (the major aspect of microbial drinking water quality) (Table 1).

## KEY FEATURES OF *LEGIONELLA* THAT MAKE IT AN APPROPRIATE INDICATOR OF WATER-BASED OPPORTUNISTIC PATHOGENS IN ENGINEERED WATER SYSTEMS

### *Legionella* Is a Major Natural Inhabitant in Engineered Water Systems and Is Listed in Water Quality Regulations

*Legionella* is a major genus of natural inhabitants in municipal EWSs (Buse et al., 2012; Wang et al., 2012a; Serrano-Suárez et al., 2013; Whiley et al., 2014a; Ashbolt, 2015a; Schwake et al., 2015; Qin et al., 2017). As a dominant OP in municipal EWSs, *Legionella* has a high concentration and a high frequency of occurrence (Wang et al., 2012a; Whiley et al., 2014a; Lu et al., 2015; Lu et al., 2016; Lu et al., 2017; Qin et al., 2017; Zhang et al., 2021b). *Legionella* contains both non-pathogenic and pathogenic species. Approximately 50% of the 61 species in *Legionella* infect humans (Borella et al., 2005a; Lau and Ashbolt, 2009; Springston and Yocavitch, 2017; The International Organization for Standardization, 2017; Gomes et al., 2018; Viasus et al., 2019; Wu et al., 2019) and significantly threaten public health (Buse et al., 2012). In this genus, *L*. *pneumophila* (*pneumophila* means “lung-loving”) is the most common life-threatening species, causing over 80% of *Legionella* infections (Reingold et al., 1984; Stout and Yu, 1997; Yu et al., 2002; Hornei et al., 2007; Falkinham, 2015; Amemura-Maekawa et al., 2018). However, most or even all species in *Legionella* are potential OPs to humans (Fields, 1996; Borella et al., 2005a; Mercante and Winchell, 2015; Lesnik et al., 2016; Salinas et al., 2021). Therefore, the presence of *Legionella* in EWSs indicates the presence of multiple clinically-relevant *Legionella* species (e.g., *L pneumophila*, *L*. *bozemanii*, *L*. *anisa*, *L*. *longbeachae*, and *L*. *londiniensis*).

Because of its implications for microbial drinking water quality and public health risks, water authorities worldwide have made guidelines, standards, and regulations to monitor and control *Legionella* in drinking water (generally not for a routine examination, which normally examines only fecal indicators) (Van Kenhove et al., 2019; Wen et al., 2020). In the US, *L*. *pneumophila* is on the Drinking Water Contaminant Candidate List 3 (CCL 3) (US EPA, 2009), the CCL 4 (US EPA, 2016), and the draft CCL 5 (US EPA, 2021). In addition, *Legionella* is the only water-based OP in the National Primary Drinking Water Regulations (US EPA, 1989). In Europe, the Netherlands, France, and Germany enforce regulatory monitoring for *Legionella* in drinking water (Ashbolt, 2015b). The revised Drinking Water Directive of the EU even requires monitoring *Legionella* in DWDSs for risk assessment (The Council of the EU, 2020). In addition, *Legionella* is a must-measure biological parameter in almost all research studies surveying microbial drinking water quality in municipal EWSs. Since *Legionella* is a major OP in municipal EWSs and is listed in multiple drinking water regulations, *Legionella* can be developed to be an indicator of microbial drinking water quality reflecting the occurrence, concentration, and behavior of OPs as a whole group in EWSs.

### *Legionella* Shares Many Critical Characteristics With Other Water-Based Opportunistic Pathogens

Water-based OPs in EWSs share several critical characteristics (Falkinham, 2015; 2020). They persist and (re)grow in EWSs, infect humans, tolerate high water temperatures, resist disinfectant residuals, and form biofilms (Figure 1). In addition, bacterial OPs such as *Legionella* resist the phagocytosis of and proliferate within their amoeba hosts (Figure 1). Because *Legionella* shares many critical characteristics with other OPs, the occurrence and concentration of *Legionella* would well indicate the occurrence and concentrations of other OPs, making *Legionella* an appropriate indicator of the public health risks posed by OPs in municipal EWSs.

#### *Legionella* Is a Major Disease Causative Biotic Agent in Municipal Engineered Water Systems

*Legionella* persists and (re)grows in EWSs (Falkinham et al., 2015b; Lu et al., 2016; Proctor et al., 2017; Wang et al., 2017; Salinas et al., 2021) and infects immunocompromised and immunocompetent populations, posing significant public health risks. *Legionella* infections are legionellosis (Borella et al., 2005a; Hornei et al., 2007), which contains community-acquired Legionnaires’ disease, outbreak-acquired Legionnaires’ disease, and Pontiac fever (Falkinham et al., 2015a). Legionnaires’ disease is a severe, life-threatening, multisystem disease involving pneumonia, while Pontiac fever is a self-limited, non-lethal, influenza-like disease (Fields et al., 2002; Lau and Ashbolt, 2009; Ashbolt, 2015a; Principe et al., 2017; Alexander et al., 2019). *Legionella* inhabits most natural and engineered aquatic environments and appears along its major reservoir, EWSs (from finished water or plant effluent to distal sites in buildings/houses) (Fields et al., 2002; Borella et al., 2005a; Whiley et al., 2014b; Falkinham et al., 2015a; Lu et al., 2015; Lu et al., 2016; Lu et al., 2017; Zhang et al., 2021b). *Legionella* in natural aquatic ecosystems (e.g., freshwater) has a low concentration and rarely infects humans (Lau and Ashbolt, 2009), while drinking water in engineered aquatic environments, especially EWSs, is the major source for legionellosis. Specifically, cooling towers, building PPSs (i.e., cold and hot water systems), household humidifiers, and pools/spas are the main contributors to legionellosis (Shands et al., 1985; Lin et al., 1998b; Hines et al., 2014; Demirjian et al., 2015; Springston and Yocavitch, 2017; Hamilton et al., 2018; Orkis et al., 2018).

*Legionella* in drinking water from municipal EWSs directly infects humans, whereas a direct transmission of legionellosis from infected persons or animals to non-infected persons is rare (US EPA, 2000; Correia et al., 2016; Beauté, 2017; Prussin et al., 2017; CDC, 2021). Drinking-water-based *Legionella* infects humans mainly through two pathways. First, inhalation of *Legionella*-containing aerosols (i.e., small droplets of drinking water in the air that contain *Legionella*) is the most common pathway (Hornei et al., 2007; Beauté, 2017; Prussin et al., 2017; CDC, 2021). For instance, immunocompromised and immunosuppressed people might get legionellosis by inhaling *Legionella*-containing aerosols generated from showerheads while showering (Brady, 1989; Schoen and Ashbolt, 2011). Therefore, showerheads are an important source of *Legionella-*containing aerosols and could be an important source of legionellosis (Cordes et al., 1981; Bollin et al., 1985; Muder et al., 1986; Collins et al., 2017; Hayes-Phillips et al., 2019). Second, aspiration or micro-aspiration of drinking water with *Legionella* (i.e., drinking water with *Legionella* accidentally goes into the lungs while drinking) is a less common pathway (Blatt et al., 1993; US EPA, 2000; Sabria and Yu, 2002; CDC, 2021). *Legionella* could also directly enter damaged skin (wound infection), but wound infection does not cause pulmonary diseases (Hornei et al., 2007).

From 2007 to 2008, 36 drinking-water-based disease outbreaks occurred in 24 states in the US and Puerto Rico, and 12 (33%) of the outbreaks were due to acute respiratory illness caused by *Legionella* (Brunkard et al., 2011). From 2009 to 2010, *Legionella* caused more outbreaks: Thirty three drinking-water-based disease outbreaks and 1,040 illnesses occurred in 17 states in the US, and *Legionella* caused 19 (58%) outbreaks and 72 (7%) illnesses (Hilborn et al., 2013). From 2011 to 2014, more than one dozen drinking-water-based disease outbreaks occurred annually in the US, resulting in hundreds of illnesses, more than 50 hospitalizations, and 6 to 7 deaths per year (Beer et al., 2015; Benedict et al., 2017). *Legionella* caused more than 50% of those outbreaks, nearly 90% of those hospitalizations, and all those deaths. The annual hospitalizations caused by community-acquired Legionnaires’ disease are from 8,000 to 18,000 in the US (Marston et al., 1997), leading to an annual hospitalization cost for water-based Legionnaires’ disease in the US to be over 433 million US dollars (Collier et al., 2012; Falkinham et al., 2015b).

#### *Legionella* Survives in a Wide Water Temperature Range in Municipal Engineered Water Systems

*Legionella* survives in a relatively wide water temperature range, and certain *Legionella* species or strains are thermotolerant or thermophilic. *Legionella* (mainly *L*. *pneumophila*) survives and/or (re)grows in a water temperature range from approximately 20 °C to approximately 50 °C (Kusnetsov et al., 1996; Leoni et al., 2001; Fields et al., 2002; Kim et al., 2002; Huang et al., 2011; Springston and Yocavitch, 2017) with an optimal (re)growth temperature range from approximately 35 °C to approximately 45 °C (Katz and Hammel, 1987; Rogers et al., 1994; Fields et al., 2002; Mathys et al., 2008; Buse and Ashbolt, 2011; Springston and Yocavitch, 2017). Certain thermophilic/thermotolerant *Legionella* species or strains, including some *L*. *pneumophila* strains, even (re)grow in hot tap water at a temperature greater than 50 °C (Mathys et al., 2008; Lesnik et al., 2016). Therefore, municipal EWSs provide a suitable water temperature range for *Legionella* (re)growth and are the major reservoir for this genus.

#### *Legionella* Has a High Resistance to Disinfectant Residuals in Municipal Engineered Water Systems

*Legionella* inherently has a high resistance to disinfectant residuals. In the US and many other countries, water utilities dose disinfectant residuals such as free chlorine, combined chlorine (mainly monochloramine), and chlorine dioxide residuals to DWDSs as a treatment barrier to suppress microbial (re)growth and prevent distribution system upsets (Olivieri et al., 1986; Norton and LeChevallier, 1997; Gagnon et al., 2004; Hwang et al., 2012; Gillespie et al., 2014; Zhang et al., 2021b). The relatively high resistance of *Legionella* to disinfectant residuals provides it with ecological advantages in municipal EWSs. Therefore, *Legionella* has a relatively high frequency of occurrence and a high concentration in EWSs, especially in seasons and/or at locations where that treatment barrier is ineffective because of disinfectant residual decay and/or consumption.

#### *Legionella* Proliferates Within Amoebae in Municipal Engineered Water Systems

*Legionella* proliferates within its amoeba hosts in municipal EWSs (Figure 1). The proliferation significantly enhances its resistance to disinfectant residuals, persistence in drinking water, and pathogenicity (Kim et al., 2002; Campos et al., 2003; Borella et al., 2005a; Lau and Ashbolt, 2009; Marciano-Cabral et al., 2010; Berjeaud et al., 2016). For instance, *L*. *pneumophila* within *A*. *polyphage* cysts survived free chlorine at a high dose (50 mg·L^−1^) for 18 h at 25 °C (Kilvington and Price, 1990). A pure planktonic *L*. *pneumophila* culture was significantly more sensitive (*p* < 0.005) to free chlorine (initial concentration 2 to 3 mg·L^−1^, contact time up to 1 h, 30 °C) and chlorine dioxide (initial concentration 0.4 mg·L^−1^, contact time up to 1 h, 30 °C) than *L*. *pneumophila* co-cultured with *Acanthamoeba* (Dupuy et al., 2011). Similarly, the *C*·*t*_99.99%_ value of free chlorine for pure planktonic *L*. *pneumophila* serogroup 1 was only 2.5 mg·min·L^−1^ (initial free chlorine concentration 0.5 mg·L^−1^, room temperature) (Cervero-Aragó et al., 2015). The same strain associated with *Acanthamoeba* was significantly more resistant to free chlorine (*C*·*t*_99.99%_ value from 19 to 245 mg·min·L^−1^; initial free chlorine concentration 0.5, 1.2, or 2.5·mg·L^−1^; room temperature). In addition, *L*. *pneumophila* concentration (log_10_^CFU^ per coupon) decreased by 1.47 to 2.07 units after exposure to free chlorine (initial concentration 0.5 mg·L^−1^, contact time 15 min to 24 h, 21 °C) in mixed-culture biofilms without *V*. *vermiformis* (Donlan et al., 2005). When *V*. *vermiformis* was present in the biofilms, *L*. *pneumophila* concentration decreased by only 0.30 to 0.90 units under the same chlorination condition. Compared with free chlorine, monochloramine (initial concentration 0.5 mg·L^−1^, contact time 15 min to 24 h, 21 °C) killed more *L*. *pneumophila* (concentration decreased by 1.77 to 2.29 units) in the biofilms without *V*. *vermiformis*. When *V*. *vermiformis* was present in the biofilms, monochloramine was less effective in killing *L*. *pneumophila* (concentration decreased by only 0.08 to 1.59 units under the same chloramination condition). Furthermore, compared with axenic planktonic *L*. *gormanii*, the resistance of *Tetrahymena pyriformis* (a ciliated protozoan) ingested *L*. *gormanii* to free chlorine (pH 7.0, 25 °C) was at least 50 folds greater (King et al., 1988). *Legionella* within amoebae is also more resistant to heavy metal ions such as copper and silver ions (Unger and Lück, 2012). Copper ions may appear in EWSs because of the use of copper or copper-containing pipes, whereas copper-silver ionization is effective in controlling *Legionella* in hospital water systems because of the strong antimicrobial activities of silver and copper ions (Liu et al., 1994; Lin et al., 1998a; Stout et al., 1998; Zhang et al., 2014; Zhang et al., 2016a; Zhang et al., 2016b). *L*. *pneumophila* in an endosymbiosis phase within *A*. *polyphage* was significantly more resistant to silver ions (0.1 mg·L^−1^), copper ions (1.0 mg·L^−1^), or the combination of silver (0.1 mg·L^−1^) and copper (1.0 mg·L^−1^) ions than a pure planktonic culture of *L*. *pneumophila* (Hwang et al., 2006).

The proliferation of *Legionella* within amoebae also enhances its tolerance to thermal treatment (Storey et al., 2004b). For instance, thermal treatment at 50 and 55 °C killed 99.99% of pure planktonic *L*. *pneumophila* serogroup 1 after 46 and 8 min, respectively (Cervero-Aragó et al., 2015). The contact time to kill 99.99% of *L*. *pneumophila* serogroup 1 associated with *Acanthamoeba* was significantly longer (745 min at 50 °C and 48 min at 55 °C). However, at 60 and 70 °C, *L*. *pneumophila* serogroup 1 associated with *Acanthamoeba* had the same tolerance to thermal treatment as the pure planktonic culture.

#### *Legionella* Forms Biofilms in Municipal Engineered Water Systems

In addition to the proliferation of *Legionella* within amoebae, biofilm formation (Figure 1) enhances the resistance of *Legionella* to disinfectant residuals (Momba et al., 2000; Wright, 2000; Donlan and Costerton, 2002; Kim et al., 2002; Loret et al., 2005; Bridier et al., 2011). For instance, the *C*·*t*_99%_ value of iodine for well-water-grown *L*. *pneumophila* serotype 1 biofilm cells (> 960 mg·min·L^−1^) was significantly greater than that for the planktonic cells of well-water-grown *L*. *pneumophila* (200 mg·min·L^−1^) (Cargill et al., 1992). Planktonic *L*. *pneumophila* took a long time (28 d) to recover in tap water amended with 0.5 mg·L^−1^ of free chlorine, whereas free chlorine at 200 mg·L^−1^ (chlorination conducted in water, contact time 1 h) inhibited only moderately the growth of one- and two-month-old *L*. *pneumophila* biofilms on stainless steel (Cooper and Hanlon, 2010). In heterotrophic drinking water biofilms, the absolute concentration of naturally occurring *L*. *pneumophila* was stable (*p* > 0.05) when free chlorine residual concentration increased from 0 to 0.2 and to 1.2 mg·L^−1^, and the relative concentration of *L*. *pneumophila* against total cell numbers even slightly increased when free chlorine residual concentration increased from 0 to 0.2 and to 1.2 mg·L^−1^ (Gião et al., 2009).

#### *Legionella* Frequently Occurs in Municipal Engineered Water Systems at a Relatively High Concentration

Because *Legionella* survives and (re)grows in a wide water temperature range, resists disinfectant residuals, proliferates within amebae, and forms biofilms, it has a high frequency of occurrence (greater than approximately 40%) and concentration in municipal EWSs (Borella et al., 2005b; Leoni et al., 2005; Wang et al., 2012a; Lu et al., 2017; Qin et al., 2017; Isaac and Sherchan, 2019; Zhang et al., 2021b). *Legionella* concentration in the bulk water in the mains of DWDSs could reach 10^7^ GCN·L^−1^ level or be even greater (10^9^ GCN·L^−1^ level at dead-ends) (Whiley et al., 2014a). In the bulk water in DWSTs, *Legionella* concentration could reach 10^4^ GCN·L^−1^ level (Qin et al., 2017). In the bulk water of PPSs, *Legionella* concentration could be greater than 10^6^ GCN·L^−1^ (Wang et al., 2012a). In the sediments of DWSTs, *Legionella* concentration could reach 10^4^ GCN·g^−1^ or 10^4^ cell equivalents·g^−1^ level (Lu et al., 2015; Qin et al., 2017). At the endpoints of PPSs (i.e., the inner surfaces of taps and showerheads), the highest concentration of *Legionella* in biofilms could be at 10^6^ GCN·swab^−1^ level (Wang et al., 2012a).

### *Legionella* Closely Correlates With Drinking Water Quality Parameters in Municipal Engineered Water Systems

#### The Close Correlations Between *Legionella* and Physicochemical Drinking Water Quality Parameters

*Legionella* often correlates with physicochemical drinking water quality parameters in municipal EWSs. For instance, in four full-scale chloraminated DWDSs in the US, *Legionella* had significant correlations with water temperature (*p* 0.002), total chlorine residual concentration (*p* 0.012), free ammonia concentration (*p* 0.037), and total THM concentration (*p* 0.019) (Zhang et al., 2021b). In a research center in Germany, *Legionella* concentration in the hot drinking water from a showerhead positively correlated with water temperature (49.2 to 57.9 °C, *R*^2^ 0.730) although *Legionella* was uncorrelated with water temperature (7.6 to 16.9 °C) in the cold drinking water from the center (*R*^2^ 0.097) (Lesnik et al., 2016). In addition, *Legionella* (*L*. *pneumophila*) commonly has a negative correlation with copper ion concentration in building hot water systems (HWSs) (*p* < 0.05) (Zacheus and Martikainen, 1994; Leoni et al., 2005) probably because copper ions have strong antimicrobial activities (Rogers et al., 1994; Borella et al., 2004; Cooper and Hanlon, 2010; Bargellini et al., 2011; Serrano-Suárez et al., 2013). *Legionella* also commonly has a negative correlation with disinfectant residual concentrations in EWSs (Zhang et al., 2021b). For instance, the concentration of *Legionella* in the tap biofilms from three laboratories in northern China was negatively associated with free and total chlorine residual concentrations in the bulk water (Liu et al., 2012). In 11 hospitals in San Antonio (Texas, USA) where *Legionella* appeared in the PPSs, average free chlorine residual concentration in patient-room tap water and the proportion of *Legionella*-positive sites negatively correlated (linear regression, *R*^2^ 0.52, *p* 0.01) (Kool et al., 1999a). Therefore, a high frequency of occurrence and a high concentration of *Legionella* in drinking water indicate that disinfectant residuals as a treatment barrier at the current levels are ineffective in suppressing OP (re)growth and the public health risks of the drinking water could be high, requiring enhanced treatment or EWS upgrade. In other words, *Legionella* could indicate the public health risks of drinking water (mainly posed by water-based OPs) in municipal EWSs.

#### The Close Correlations Between *Legionella* and Microbial Drinking Water Quality Parameters

*Legionella* in EWSs closely correlates with microbial drinking water quality parameters. In general, *Legionella* positively correlates with major OPs such as *Mycobacterium*, *Pseudomonas* (especially *P*. *aeruginosa*), *V*. *vermiformis*, and *Acanthamoeba* in EWSs (Wang et al., 2012a; Serrano-Suárez et al., 2013; Lu et al., 2016). For instance, in the bulk water of four full-scale chloraminated DWDSs in the US, the concentration of *Legionella* significantly and positively correlated with those of *Mycobacterium* (*p* 0.042), *Pseudomonas* (*p* < 0.001), and total OPs (*p* < 0.001) (Zhang et al., 2021b).

In conclusion, *Legionella* closely correlates with multiple physicochemical and microbial drinking water quality parameters in municipal EWSs. The close correlations between *Legionella* and other OPs suggest that the presence and concentration of *Legionella* could infer the presence and concentrations of other major OPs. The close correlations between *Legionella* and physicochemical drinking water quality parameters (such as disinfectant residuals) further suggest that *Legionella* could indicate the overall drinking water quality and the potential public health risks posed by OPs in EWSs.

### *Legionella* Indicates the Efficacies of Disinfectant Residuals and Explains Their Modes of Action in Engineered Water Systems

#### *Legionella* Indicates the Efficacies of Disinfectant Residuals in Engineered Water Systems

Disinfectant residuals at relatively high levels are effective in preventing the (re)growth of OPs in EWSs (Olivieri et al., 1986; Norton and LeChevallier, 1997; Gagnon et al., 2004; Hwang et al., 2012; Gillespie et al., 2014; Zhang and Lu, 2021). For instance, *Legionella* (*L*. *pneumophila*) was absent from the bulk water (finished water, DWSTs, water mains entering hospitals, and taps) of an EWS (Pennsylvania, USA) because free chlorine residual of approximately 0.2 mg·L^−1^ appeared throughout the system (States et al., 1987). The frequencies of occurrence of *Legionella* in tap water (26%) and water-main biofilms (0%) in a chloraminated EWS in the US were significantly lower than those (64% for tap water and 43% for biofilms) in a no-residual EWS in Norway (Waak et al., 2018). In the effluents of simulated household water heaters, *Legionella* concentration negatively correlated with the concentrations of disinfectant residuals (free chlorine or chloramines) in the influents of the heaters (Spearman’s rank correlation coefficients between −0.752 and −0.019) (Wang et al., 2015). Similarly, the concentration of *Legionella* in the bulk water of simulated DWDSs (sampled 14 months after the start-up) negatively correlated with chloramine residual concentrations (Spearman’s rank correlation coefficients between −0.908 and −0.693, *p* < 0.01) (Wang et al., 2012b). In the hot water from 67 buildings in Finland, chlorine residual inhibited the (re)growth of *Legionella* (Spearman’s rank correlation coefficient between chlorine residual level and *Legionella* concentration −0.19, *p* < 0.01) (Zacheus and Martikainen, 1994). A recent study found that the distal site/outlet (hot water) positivity of *Legionella* for 28 buildings in the US was uncorrelated with free chlorine residual level in the incoming cold water of the buildings (Pierre et al., 2019). However, free chlorine residual still prevented the (re)growth of *Legionella* in those buildings: Cold water from water mains entering the buildings (free chlorine residual 0.34 mg·L^−1^, *Legionella* concentration 1.4 × 10^4^ CFU·L^−1^, *Legionella* positivity 3.7%) and cold water storage tanks (free chlorine residual 0.23 mg·L^−1^, *Legionella* concentration 3.8 × 10^3^ CFU·L^−1^, *Legionella* positivity 10.3%) had much greater free chlorine residual concentration than hot water from hot water return lines (free chlorine residual 0.07 mg·L^−1^, *Legionella* concentration 3.6 × 10^5^ CFU·L^−1^, *Legionella* positivity 26.7%) and hot water distal outlets (free chlorine 0.05 mg·L^−1^, *Legionella* concentration 4.2 × 10^5^ CFU·L^−1^, *Legionella* positivity 30.4%) and thus much lower *Legionella* concentration and positivity.

Since disinfectant residuals are effective in inhibiting the (re) growth of OPs in EWSs, injecting disinfectants into building water systems reduces the frequencies of occurrence and concentrations of OPs. For instance, the positivity of *Legionella* at the cold and hot water distal outlets significantly decreased from approximately 60% to less than 10% after two hospitals in the US injected chlorine dioxide into the incoming water mains (Zhang et al., 2009). Likewise, after a hospital (Pennsylvania, USA) injected monochloramine into the HWS of a building, the distal site/outlet positivity of culturable *Legionella* was significantly lower compared with the control building (no monochloramine dosing) or the same building before the chloramination (Baron et al., 2014; Duda et al., 2014; Baron et al., 2015). In a hospital building (Modena, Italy), injecting monochloramine into a HWS (target concentration 1.5 to 3.0 mg·L^−1^, continuous application) significantly reduced the frequencies of occurrence of *L*. *pneumophila* in the hot water from heaters, return loops, and distal outlets from 97.0% (of 32 samples) to 13.3% (of 60 samples) (first-year application) (Marchesi et al., 2011; Marchesi et al., 2012). Meanwhile, the geometric means of *L*. *pneumophila* concentration for the positive hot water samples dramatically dropped from 1.7 × 10^4^ to 3.4 × 10^2^ CFU·L^−1^ after the dosing of monochloramine (first-year application). Over three years after the dosing of monochloramine, *Legionella* appeared in only 8 hot water samples (9.5% of 84 samples) with a low concentration (3.3 × 10^2^ CFU·L^−1^, geometric mean for the 8 positive samples) (Marchesi et al., 2013).

As discussed above, because *Legionella* concentration has a general negative correlation with disinfectant residual levels, *Legionella* well indicates the efficacy of an important treatment barrier in municipal EWSs, secondary disinfection (i.e., dosing and maintaining disinfectant residuals) (Zhang et al., 2021b). When *Legionella* occurs in EWSs at a high frequency and/or a high concentration, the treatment barrier is ineffective in preventing the (re)growth of *Legionella* and other OPs, where the OPs might have a high potential causing disease (such as legionellosis) outbreaks. Therefore, *Legionella* occurring at a high frequency and/or concentration indicates drinking water quality deterioration and requires enhanced treatment (such as booster disinfection) and/or EWS cleaning/upgrade. Oppositely, drinking water from an EWS where *Legionella* occurs at a low frequency and/or a low concentration generally has a high quality and will less likely cause disease outbreaks due to the presence of OPs.

#### Monochloramine Is More Effective Than Free Chlorine in Controlling *Legionella* in Engineered Water Systems

Monochloramine has a long-lasting bactericidal effect and is more effective than free chlorine in controlling *Legionella* in municipal EWSs (Kool, 2001; Lin et al., 2011; Buse et al., 2012; Springston and Yocavitch, 2017). Therefore, the prevalence of *Legionella* in chloraminated EWSs is lower than that in chlorinated EWSs, suggesting that drinking water from chloraminated EWSs is less likely to cause outbreaks of legionellosis (Kool, 2001; Kim et al., 2002; Lin et al., 2011; Buse et al., 2012). Among 32 hospitals in the US where drinking-water-associated, hospital-acquired Legionnaires’ disease outbreaks occurred at least once per hospital from 1979 to 1997, free chlorine was the disinfectant residual in the drinking water for 31 hospitals, and monochloramine was the disinfectant residual in the drinking water for only one (Kool et al., 1999b; Kool et al., 2000). By contrast, for 48 hospitals in the US without a drinking-water-associated, hospital-acquired Legionnaires’ disease outbreak from 1979 to 1997, free chlorine was the disinfectant residual for 36 hospitals, and monochloramine was the disinfectant residual for 12. Therefore, hospitals supplied with drinking water with monochloramine residual (adjusted odds ratio for outbreaks of Legionnaires’ disease 1.0) are significantly less likely to have drinking-water-associated, hospital-acquired Legionnaires’ disease outbreaks than hospitals with free chlorine residual in drinking water (adjusted odds ratio for outbreaks of Legionnaires’ disease 10.2). Similarly, 16% of 38 hospitals in the US that had outbreaks/cases of hospital-acquired Legionnaires’ disease used drinking water with monochloramine residual, while 46% of 114 hospitals in the US without an outbreak/case of hospital-acquired Legionnaires’ disease used drinking water with monochloramine residual (Heffelfinger et al., 2003). As a result, the adjusted odds ratio (0.20) for definite cases or outbreaks of hospital-acquired Legionnaires’ disease in a hospital using drinking water with monochloramine residual is low.

#### *Legionella* Explains the Modes of Action of Free Chlorine and Monochloramine Residuals in Engineered Water Systems

Free chlorine has a higher oxidation-reduction (redox) potential than monochloramine and is thus a stronger oxidant and biocide (Table 2) (Akin et al., 1982; Wolfe et al., 1984; Kouame and Haas, 1991; Gagnon et al., 2004; Lee et al., 2011b; Chiao et al., 2014; Zhang et al., 2018). Therefore, free chlorine is more effective than monochloramine in killing planktonic *Legionella*. For instance, the *C*·*t*_99.98% to 99.99%_ values of free chlorine (1 to 5 mg·min·L^−1^, room temperature or 30 °C) against planktonic *L*. *pneumophila* (Dupuy et al., 2011; Cervero-Aragó et al., 2015) were significantly lower than the *C*·*t*_99.9% to 99.99%_ values of monochloramine (16 to 65 mg·min·L^−1^, 25 to 35 °C) against planktonic *L*. *pneumophila* (Dupuy et al., 2011; Jakubek et al., 2013).

However, monochloramine residual is more effective than free chlorine residual in controlling *Legionella* in municipal EWSs. The greater effectiveness of monochloramine residual in preventing *Legionella* (re)growth in EWSs is due to three reasons. First, the majority of *Legionella* cells in EWSs are in biofilms/sediments and within protozoans (e.g., amoebae) (Stout et al., 1985; Lin et al., 1998b; Fields et al., 2002; Batté et al., 2003; Lau and Ashbolt, 2009; Abdel-Nour et al., 2013; Ashbolt, 2015a; Falkinham, 2015). Approximately 95% of the biomass in EWSs is in biofilms and sediments on pipe walls, and only 5% of the biomass is in bulk water (Block, 1992; Flemming et al., 2002; Wingender and Flemming, 2011). For instance, in a pilot-scale HWS, more than 98.5% of *Legionella* cells lived in the pipe biofilms (Saby et al., 2005). Monochloramine well penetrates biofilms and controls biofilm contamination (LeChevallier et al., 1988; Norton and LeChevallier, 1997; Lee et al., 2011b; Pressman et al., 2012). By contrast, the rapid reaction between free chlorine and organic matter of biofilm origin greatly prevents free chlorine from penetrating biofilms (Chen and Stewart, 1996; Grobe et al., 2002; Bridier et al., 2011). For example, exposing a 526 μm thick *P*. *aeruginosa* biofilm to a flowing free chlorine solution (18.6 mg·L^−1^) for 3 h only raised free chlorine concentration at the substratum to 1.8 mg·L^−1^ (i.e., approximately 10% of the bulk free chlorine level) (Chen and Stewart, 1996). Similarly, free chlorine level in dual-species biofilms of *P*. *aeruginosa* and *K*. *pneumoniae* was only 20% or less of the free chlorine level in the bulk liquid (De Beer et al., 1994). Therefore, monochloramine residual penetrates biofilms faster and more completely than free chlorine residual in municipal EWSs (Lee et al., 2018). Indeed, the maximum initial penetration rate of monochloramine to nitrifying biofilms was approximately 170 times that of free chlorine (equivalent chlorine levels) (Lee et al., 2011b). Because monochloramine penetrates biofilms faster and more completely but free chlorine only inactivates cells on biofilm surfaces contacting bulk water (Kelsey, 2014; Lee et al., 2018), monochloramine is more effective in killing biofilm cells of *Legionella*. For instance, monochloramine and free chlorine at the same *C*·*t* value of 270 mg·min·L^−1^ (initial concentration 1.5 mg·L^−1^ for both disinfectants) reduced *L*. *pneumophila* concentration in mixed-culture bacterial biofilms by approximately 99.7% and approximately 87.4%, respectively (Donlan et al., 2001). In addition, monochloramine is more effective than free chlorine in killing biofilm cells of other bacterial species: The *C*·*t*_> 99.9%_ value of monochloramine (3 mg·min·L^−1^) against *K*. *pneumoniae* attached to glass slides was significantly lower than the *C*·*t*_99.6%_ value of free chlorine (150 mg·min·L^−1^) (LeChevallier et al., 1988). The slow and incomplete biofilm penetration of free chlorine is an important reason for the relatively strong resistance of biofilm cells to free chlorine (Grobe et al., 2002).

Second, the association between *Legionella* and its amoeba hosts significantly enhances the resistance of *Legionella* to free chlorine (Cervero-Aragó et al., 2015) rather than monochloramine probably because monochloramine penetrates amoebae more completely. For instance, free chlorine with a *C*·*t* value of 5 mg·min·L^−1^ (30 °C) killed 99.98% of pure planktonic *L*. *pneumophila* but only 98.74% of *L*. *pneumophila* co-cultured with *Acanthamoeba* (*p* < 0.005) (Dupuy et al., 2011). However, monochloramine (*C*·*t* value 2 mg·min·L^−1^, 30 °C) was comparably effective (*p* > 0.005) in killing pure planktonic *L*. *pneumophila* and *L*. *pneumophila* co-cultured with *Acanthamoeba*. In addition, monochloramine is more effective in killing free-living amoebae (i.e., *Acanthamoeba*, *Naegleria*, and *Vermamoeba*) than free chlorine (Dupuy et al., 2014) although opposite evidence does exist (De Jonckheere and Van de Voorde, 1976; Ercken et al., 2003; Loret and Greub, 2010; Mogoa et al., 2011). Therefore, monochloramine is more effective than free chlorine in killing amoeba-associated *Legionella*. For instance, monochloramine at 0.5 mg·L^−1^ killed 84.51% (contact time 3 h) or 97.43% (contact time 24 h) of biofilm-associated *L*. *pneumophila* in the presence of *V*. *vermiformis* (Donlan et al., 2005). Free chlorine at 0.5 mg·L^−1^ killed much less biofilm-associated *L*. *pneumophila* in the presence of *V*. *vermiformis* at a contact time of 3 h (78.62%) or 24 h (87.41%).

Third, since free chlorine is more reactive and has a higher redox potential (Table 2) than monochloramine, monochloramine residual is more stable and easier to maintain in EWSs (Wolfe et al., 1984; Cunliffe, 1990; Vikesland et al., 1998; Vikesland and Valentine, 2000; Kim et al., 2002; Dupuy et al., 2011; Gall et al., 2015; Zhang et al., 2018). Moreover, monochloramine has a wider working pH range than free chlorine in water (Ercken et al., 2003; Lin et al., 2011; Springston and Yocavitch, 2017; Baron et al., 2019).

### Numerous Methods Are Available to Detect and Quantify *Legionella* in Engineered Water Systems

Numerous methods are available to recover, identify, and/or quantify *Legionella* in drinking water from EWSs. Those methods include the “gold standard” culture-based assays (e.g., with special media or amoebal co-culture), molecular assays through analyzing DNA/RNA/proteins (e.g., qPCRs, high-throughput DNA sequencing, and flow cytometry), and phenotypic assays (e.g., immunoassays) (Lee et al., 2011a; Aw and Rose, 2012; Wang et al., 2017; Zhang et al., 2021a).

Among the various available *Legionella* detection, recovery, and quantification assays, standard culture-based methods and (regular or traditional) qPCRs are the most widely used (Whiley and Taylor, 2016). Standard culture-based assays as the gold standard detect only culturable *Legionella* in drinking water (Marinelli et al., 2017). However, *Legionella* and other bacteria, including other OPs, in (chloraminated/chlorinated) EWSs could and often enter a VBNC state (Alleron et al., 2008; Kirschner, 2016; Casini et al., 2018; Cullom et al., 2020; Sciuto et al., 2021). VBNC *Legionella* is alive and infective and produces virulence proteins (or, at least retains its virulence) (Oliver, 2010; Alleron et al., 2013; Dietersdorfer et al., 2018). However, a standard culture-based approach cannot detect or recover VBNC *Legionella* (Marinelli et al., 2017; Whiley, 2017; Párraga-Niño et al., 2018). Therefore, standard or traditional culture-based assays underestimate the prevalence and public health risks of *Legionella* in municipal drinking water. On the other hand, traditional or regular qPCRs as a very common method for *Legionella* detection and quantification in EWSs detect total DNA or genetic markers of *Legionella*, including those from both live (culturable and VBNC) and dead *Legionella* cells (Dusserre et al., 2008; Wang et al., 2012a; Whiley et al., 2014a; Taylor et al., 2014; Lu et al., 2015; Lu et al., 2016; Lu et al., 2017; Qin et al., 2017; Waak et al., 2018; Isaac and Sherchan, 2019; Zhang et al., 2021b). Therefore, traditional qPCRs (significantly) overestimate the health risks of *Legionella* in EWSs because dead *Legionella* cells no longer infect humans (Fleischmann et al., 2021). One promising approach to resolving this overestimation issue is propidium monoazide qPCRs (PMA-qPCRs). PMA is a DNA intercalating molecule that penetrates only membrane-damaged or membrane-compromised cells (i.e., dead cells), binds to DNA within the dead cells, and inhibits DNA amplification during subsequent qPCRs (Nocker et al., 2007; Li and Chen, 2013; Golpayegani et al., 2019; Guo et al., 2021). The DNA or genetic markers of *Legionella* within membrane-intact (i.e., live, including culturable and VBNC) cells are not bound to PMA and are normally amplified during qPCRs. Therefore, PMA-qPCRs detect only live (culturable and VBNC) *Legionella* cells and more accurately estimate the public health risks of *Legionella*. Researchers have successfully applied PMA-qPCRs to detect live *Legionella* (*L*. *pneumophila*) cells in various settings including drinking water (Yáñez et al., 2011; Slimani et al., 2012; Li et al., 2015; Bonetta et al., 2017; Bonetta et al., 2018; Kontchou and Nocker, 2019). Using PMA-qPCRs to detect *Legionella* (and other microbes) has certain limitations such as incomplete reduction/suppression of the qPCR signals of DNA from dead cells, especially when qPCR amplicons are short or when the abundance of dead cells is high (Pan and Breidt, 2007; Kralik et al., 2010; Luo et al., 2010; Yáñez et al., 2011; Taylor et al., 2014; Ditommaso et al., 2015; Codony et al., 2020). In addition to PMA-qPCRs, reverse transcription qPCRs (RT-qPCRs) are a useful tool for detecting viable *Legionella* in (drinking) water (Bej et al., 1991; Boss et al., 2018).

A comprehensive summary of the traditional and novel *Legionella* recovery, identification, and quantification methods is beyond the scope of this literature review. Interested readers may consult a few publications for the details of those methods (Marre et al., 2001; Fields, 2007; Buse et al., 2012; Kirschner, 2016; Whiley and Taylor, 2016; Wang et al., 2017; Párraga-Niño et al., 2018; Baume et al., 2019; Ezenarro et al., 2020; Walker and McDermott, 2021). Because of its frequent occurrence, relatively high concentration, and various quantification methods, *Legionella* is a promising indicator of water-based OPs (the major aspect of microbial drinking water quality) in municipal EWSs.

## DISCUSSION AND FUTURE WORK

Our early work preliminarily proposed that *Legionella* could be an indicator of non-fecal pathogens in municipal DWDSs (Lu et al., 2016). Our recent study examined the dynamics of dominant OPs in four full-scale DWDSs and confirmed that *Legionella* is a promising indicator of water-based OPs and microbial drinking water quality in municipal EWSs (Zhang et al., 2021b). The current systematic review greatly expands our previous argument and provides convincing reasons for the appropriateness of *Legionella* as an indicator of water-based OPs (the major aspect of microbial drinking water quality) in municipal EWSs. However, this review does not imply that *Legionella* should be used as a sole indicator to infer microbial drinking water quality (Huang et al., 2021). First, *Mycobacterium* is highly resistant to disinfectant residuals (Falkinham, 2015) and could be more resistant to chloramines than *Legionella* (Lin et al., 2011). Therefore, in a chloraminated EWS, a low frequency of occurrence and a low concentration of *Legionella* do not necessarily indicate a low health risk from *Mycobacterium*. In this case, water utilities might use *Mycobacterium* and *Legionella* in combination to better examine microbial drinking water quality (Lu et al., 2017; Huang et al., 2021). Future work needs to comprehensively compare the appropriateness of *Legionella* and *Mycobacterium* as indicators of water-based OPs and microbial drinking water quality. Evaluating the combined use of *Legionella* and *Mycobacterium* as indicators of microbial water quality is also necessary. Second, this review does not imply that we shall abandon the routine monitoring of conventional fecal indicators in municipal EWSs. Even though conventional fecal indicators do not indicate the presence and concentrations of OPs in drinking water, they do well indicate issues in treatment utilities (e.g., failure of disinfection processes), EWS problems (i.e., infiltration, backflow, and water main breaks), and the presence of enteric pathogens and/or fecal contaminants in drinking water. Therefore, this systematic review does not argue substituting *Legionella* for conventional fecal indicators as the sole indicator of microbial drinking water quality. Instead, this review argues that *Legionella* as an additional or supplementary indicator for microbial water quality should be used in conjunction with conventional fecal indicators. The combined use of *Legionella* and fecal indicators as microbial drinking water quality indicators would help more comprehensively understand the health risks of drinking water in municipal EWSs. Future work needs to explore how to use *Legionella* and conventional fecal indicators in combination to better monitor microbial drinking water quality and better protect public health.

This review focuses on convincing the water industry that *Legionella* is an appropriate supplementary indicator microorganism inferring the prevalence of water-based OPs (the major aspect of microbial drinking water quality) in municipal EWSs. When an indicator microorganism is proposed, carefully selecting a threshold (i.e., a numerical permissible limit) of the indicator that indicates a health risk or requires an action to be taken is important. For instance, when the concentration of the indicator exceeds a certain threshold, a boil-water-advisory should be issued, and enhanced treatment (i.e., booster disinfection) is required. Future work should propose *Legionella* limits (i.e., concentration limits and positivity limits) for drinking water that indicate serious public health risks and require enhanced or additional treatment.

This review grouped municipal DWDSs and building PPSs to EWSs for the ease of discussion. However, microbial growth conditions between DWDSs and PPSs are significantly different (Zhang et al., 2021b). In addition, the legal landscapes of water responsibility between DWDSs and PPSs are distinct. For instance, general microbial water quality monitoring is typically done only in DWDSs rather than in PPSs. *Legionella* as an indicator of microbial water quality in those two systems thus has distinct roles. Future work needs to clarify how to appropriately use *Legionella* as an indicator of water-based OPs and microbial water quality in these two different systems.

## CONCLUSION

*Legionella* as an important natural inhabitant frequently occurs in municipal EWSs with a relatively high concentration. Opportunistically pathogenetic species in the genus *Legionella* (mainly *L*. *pneumophila* but include many other species) in EWSs frequently cause drinking-water-based disease (e.g., Legionnaires’ disease) outbreaks and pose significant public health risks. Quantification of *Legionella* in EWSs with modern molecular-based assays (especially qPCRs) is affordable and fast. *Legionella* in EWSs closely correlates with major physicochemical and microbial water quality parameters, especially the occurrence and concentrations of water-based OPs. Biofilms/sediments and amoebae are the main niches of *Legionella* in EWSs, and the association of *Legionella* with biofilms/sediments and amoebae significantly enhances the already high resistance of *Legionella* to disinfectant residuals. *Legionella* indicates the efficacies and explains the modes of action of disinfectant residuals in EWSs. For instance, monochloramine is more effective than free chlorine in controlling *Legionella* in EWSs because monochloramine penetrates biofilms/sediments/amoebae faster and more completely and has a long-lasting antimicrobial/bactericidal effect (i.e., more stable than free chlorine). Those features of *Legionella* make it a promising indicator of the prevalence of water-based OPs and microbial water quality in municipal EWSs.

## Figures and Tables

**TABLE 1 |:** A brief comparison for the appropriateness of common natural inhabitants in municipal engineered water systems (EWSs) as indicators of opportunistic pathogens (OPs).

Natural inhabitant	Main characteristics	Appropriate as an indicator of OPs?
Non-pathogenic species	Abundant in EWSs; Harmless to humans; Occurrence and concentrations do not correlate with the public health risks posed by OPs	No
*P. aeruginosa, Aeromonas, A. baumannii, Flavobacterium, Acanthamoeba, V. vermiformis,* and *N. fowleri*	Low frequencies of occurrence; Low concentrations; Sometimes absent; The health risks of drinking water could be still high when they appear in EWSs at low frequencies and/or low concentrations because *Legionella* and *Mycobacterium* as the most dominant OPs could occur at high concentrations at the same time	No
*Mycobacterium*	High frequency of occurrence; High concentration; Very high resistance to disinfectant residuals; When disinfectant residuals are at levels effective enough in suppressing the (re) growth of OPs (as well as enteric pathogens and/or fecal contaminants) and ensuring a good drinking water quality, the concentration of *Mycobacterium* could be still relatively high	Could be a potential indicator of OPs but not as good as *Legionella*
*Legionella*	High frequency of occurrence; High concentration; The predominant biotic disease causative agent in EWSs; High resistance to disinfectant residuals; Closely correlates with more physicochemical and microbial water quality parameters than *Mycobacterium*; Its high (low) concentration well indicates high (low) public health risks of drinking water	Might be the best candidate for an indicator of water-based OPs and microbial drinking water quality

**TABLE 2 |:** The oxidation-reduction (redox) potentials of free chlorine and monochloramine.

Disinfectant	Redox couple (1 e^−^)	Half-reaction	*E’* (V)
Free chlorine	$\frac{1}{2}{\text{Cl}}_{2(\text{aq})}/{\text{Cl}}_{\left(\text{aq}\right)}^{-}$	$\frac{1}{2}{\text{Cl}}_{2(\text{aq})}+{\text{e}}^{-}\to {\text{Cl}}_{\left(\text{aq}\right)}^{-}$	1.397
Free chlorine	$\frac{1}{2}{\text{Cl}}_{2(\text{g})}/{\text{Cl}}_{\left(\text{aq}\right)}^{-}$	$\frac{1}{2}{\text{Cl}}_{2(\text{g})}+{\text{e}}^{-}\to {\text{Cl}}_{\left(\text{aq}\right)}^{-}$	1.361
Free chlorine	$\frac{1}{2}{\text{HOCl}}_{\text{(aq) }}/\frac{1}{2}{\text{Cl}}_{\text{(aq) }}^{-}$	$\frac{1}{2}{\text{HOCl}}_{\left(\text{aq}\right)}+\frac{1}{2}{\text{H}}_{\left(\text{aq}\right)}^{+}+{\text{e}}^{-}\to \frac{1}{2}{\text{H}}_{2}{\text{O}}_{\left(1\right)}+\frac{1}{2}{\text{Cl}}_{\left(\text{aq}\right)}^{-}$	1.288
Monochloramine	$\frac{1}{2}{\text{NH}}_{2}{\text{Cl}}_{\text{(aq) }}/\frac{1}{2}{\text{Cl}}_{\text{(aq) }}^{-}$	$\frac{1}{2}{\text{NH}}_{2}{\text{Cl}}_{\left(\text{aq}\right)}+{\text{H}}_{\left(\text{aq}\right)}^{+}+{\text{e}}^{-}\to \frac{1}{2}{\text{NH}}_{4(\text{aq})}^{+}+\frac{1}{2}{\text{Cl}}_{\left(\text{aq}\right)}^{-}$	1.097

*Source:* Zhang et al. (2018). E*’: The redox potential (versus the standard hydrogen electrode) for the half-reaction under biochemical standard state conditions (pH 7*.*0, 25 °C, 101,325 Pa)*.
